# Risk factor-dependent dynamics of atopic dermatitis: modelling multi-scale regulation of epithelium homeostasis

**DOI:** 10.1098/rsfs.2012.0090

**Published:** 2013-04-06

**Authors:** Elisa Domínguez-Hüttinger, Masahiro Ono, Mauricio Barahona, Reiko J. Tanaka

**Affiliations:** 1 Department of Bioengineering, Imperial College London, London SW7 2AZ, UK; 2 Department of Mathematics, Imperial College London, London SW7 2AZ, UK; 3 Institute of Child Health, University College London, 30 Guilford Street, London WC1N 1EH, UK

**Keywords:** multi-scale modelling, epithelium, time-scale separation, atopic dermatitis, risk factors

## Abstract

Epithelial tissue provides the body with its first layer of protection against harmful environmental stimuli by enacting the regulatory interplay between a physical barrier preventing the influx of external stimuli and an inflammatory response to the infiltrating stimuli. Importantly, this interdependent regulation occurs on different time scales: the tissue-level barrier permeability is regulated over the course of hours, whereas the cellular-level enzymatic reactions leading to inflammation take place within minutes. This multi-scale regulation is key to the epithelium's function and its dysfunction leads to various diseases. This paper presents a mathematical model of regulatory mechanisms in the epidermal epithelium that includes processes on two different time scales at the cellular and tissue levels. We use this model to investigate the essential regulatory interactions between epidermal barrier integrity and skin inflammation and how their dysfunction leads to atopic dermatitis (AD). Our model exhibits a structure of dual (positive and negative) control at both cellular and tissue levels. We also determined how the variation induced by well-known risk factors for AD can break the balance of the dual control. Our model analysis based on time-scale separation suggests that each risk factor leads to qualitatively different dynamic behaviours of different severity for AD, and that the coincidence of multiple risk factors dramatically increases the fragility of the epithelium's function. The proposed mathematical framework should also be applicable to other inflammatory diseases that have similar time-scale separation and control architectures.

## 1. Introduction

Most physiological processes are tightly controlled by regulatory interactions across different temporal and spatial scales. An important example of such multi-scale regulatory interactions is found in the epithelium, the tissue covering the organs and cavities of animal bodies. The epithelium provides the first layer of protection against harmful environmental stimuli, such as bacteria, chemicals or pollen, through a tightly controlled interplay between the regulation of the physical barrier permeability to the environmental stimuli and the immune reaction to the infiltrating stimuli [1–4]. Importantly, while the regulation of the physical barrier permeability results from the orchestration between growth, differentiation and apoptosis of different cell types of the order of hours or days [5,6], the phenotype of each cell within the epithelium is determined locally based on the concentration of local effectors (such as enzymes and gene transcripts) regulated through protein–protein interaction (PPI) networks of the order of minutes [7,8]. In turn, the strength of the inter-epithelial stimulus depends on epithelial permeability, a tissue-level property determined by the epithelium's homeostasis. Therefore, regulation of epithelium function involves an interplay of fast inflammation-inducing PPIs with slow barrier-reconfiguring processes in an archetypical example of multiple-scale regulatory feedback.

Defects in this multi-scale feedback regulation may lead to the loss of epithelial homeostasis, inflammation and the development of diseases, including atopic diseases, such as asthma [3], allergic rhinitis (hay fever) [9] and atopic dermatitis (AD) [10]. These diseases are typically characterized by two strongly interconnected symptoms (loss of barrier function and exacerbated inflammatory reactions to environmental stimuli) that occur at different spatio-temporal scales.

Epidemiological and biochemical studies have established the relevance of a variety of risk factors that predispose individuals to atopic diseases, although the exact causes of these diseases are not fully understood. Risk factors include genetic polymorphisms, as well as environmental factors (such as exposure to allergens or pollutants) [11–14]. However, the role of individual risk factors in epithelial function is difficult to pinpoint experimentally because of the complexity of the highly interconnected, multi-scale epithelium regulatory network. Indeed, loss of regulation triggered by a risk factor may be the result of imbalances between different processes across cellular and tissue levels. Furthermore, clinically relevant studies are hampered by the interference between multiple risk factors, often observed in patients with atopic diseases [15]. This paper presents a mathematical framework to analyse systems-level mechanisms for atopic diseases. Our framework allows us to systematically assess the effects of single or multiple risk factors on epithelium function; study how the coincidence of multiple risk factors severely increases the propensity to develop atopic diseases; and deepen our understanding of the underlying mechanisms that lead to pathological conditions.

Several mathematical models have been proposed for studying the *spatial dynamics* of epithelial cells, including migration, proliferation, differentiation and death of gut cells in relation to colorectal cancer [16,17], or reorganization of the epidermis during wound healing [18,19]. However, these models do not explicitly consider the regulatory interplay between the two defensive mechanisms of the epithelium, although the skin barrier integrity and the inflammatory response mediated by PPIs have been modelled in isolation [20,21]. Our framework builds upon a mathematical model of the *cellular-level* regulatory mechanisms leading to AD that focuses on the activity of proteolytic enzyme kallikreins (KLKs) responsible for both skin desquamation and inflammation [22]. Although this cellular model successfully captures key clinical features of AD and their dependence on some risk factors, it does not consider the intertwined, multi-scale feedback regulation across cellular and tissue levels.

This paper proposes an ordinary differential equation (ODE) model of epithelium function that includes the essential regulatory interplay between the two protective properties that operate on different time scales: physical barrier integrity (on slow scales) and immune reactions to eradicate the stimulus (on fast scales). We focus on the disruption of homeostasis in the epidermis leading to AD, an archetypical example of atopic diseases that affects nearly 30 per cent of the paediatric population worldwide [14]. AD is characterized by an abnormal hypersensitivity to environmental stimuli and a loss of skin barrier function and is commonly manifested as dry, scaly skin and a rash. Note, too, that the essential features of complex regulatory mechanisms clarified by our multi-scale model can be used to broaden our understanding of other pathophysiological processes related to the loss of epithelial homeostasis, particularly in asthma and hay fever, given the confirmed association between AD and other atopic diseases [14].

**Figure 1.**
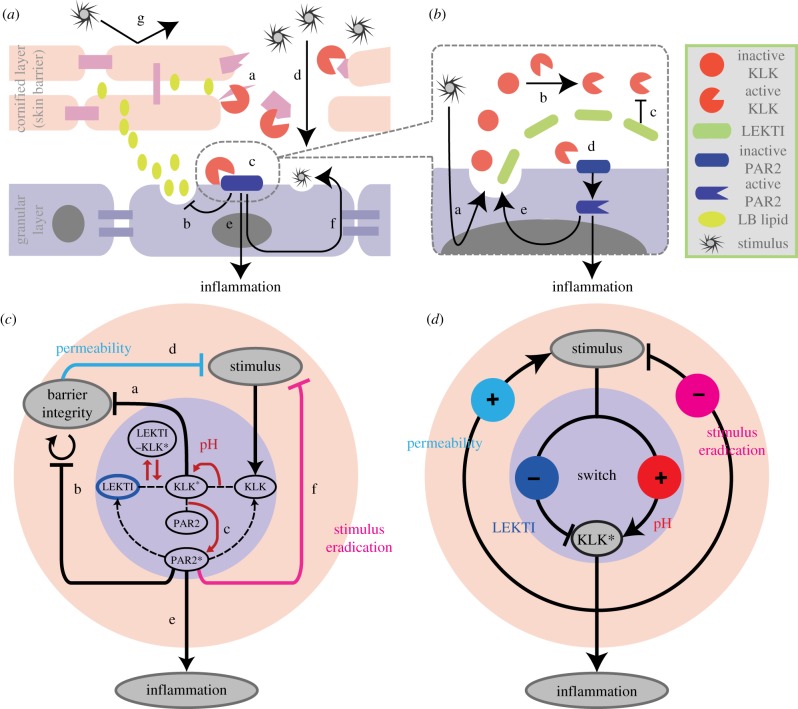
Multi-scale model of AD. (*a*) Tissue-level reactions considered. (a) Skin desquamation by KLK* (active KLK), (b) inhibition of LB lipid release by PAR2* (active PAR2), (c) PAR2 activation by KLK* (at the cellular level), (d) penetration of the environmental stimuli into the inner layers of the epidermis, (e) inflammation triggered by PAR2* (at the cellular level), (f) eradication of stimuli by immune reactions, and (g) protection against environmental stimuli by healthy skin barrier. (*b*) Cellular-level reactions considered. (a) Release of inactive KLK and its inhibitor LEKTI enhanced by inter-epidermal stimulus, (b) auto-activation of KLK by proteolysis, (c) inhibition of KLK* by LEKTI, (d) activation of PAR2 by KLK*, leading to inflammation, and (e) increased release of KLK and LEKTI upon PAR2 activation. (*c*) Schematic representation of the multi-scale model for epidermal function, consisting of the tissue-level regulation for skin barrier integrity (beige circle) and the cellular-level PPI leading to inflammation (violet circle). (*d*) Core structure of our model and the risk factors. Switch behaviour of inflammation occurs through stimulus-induced PPI at the cellular level (violet circle) mediated by a combination of positive (KLK* activation) and negative (KLK* inhibition by LEKTI) controls. The concentration of the inter-epidermal stimulus is controlled at the tissue level (beige circle) by a combination of positive (via skin barrier permeability) and negative (via stimulus eradication) feedbacks from KLK*-activated PAR2* leading to inflammation. We consider four risk factors for AD, ‘high pH’, ‘low LEKTI’, ‘high permeability’ and ‘weak stimulus eradication’, each of which breaks the balanced control at the cellular and tissue levels.

Crucially, it is the slow, tissue-level behaviour that is observable and used for treatment in clinical settings, yet such long-term dynamics are intricately interlinked with the fast, cellular PPIs. To study the slow dynamics, we assume a separation of time scales and consider quasi-stationarity at the cellular level, leading to a system of differential–algebraic equations. Our model (figure 1) is constructed based on the view that overall homeostasis is achieved by mainly two types of regulated balance: one between activation and inhibition of KLKs at the cellular level and another between positive and negative feedbacks from inflammation-inducing signalling pathways to the strength of stimulus at the tissue level. The interplay between the fast cellular-level reactions [22] and the much slower tissue-level dynamics controlling the concentration of the inter-epidermal stimulus is needed in order to understand AD as being a result of the disruption of epithelium homeostasis: excessive skin desquamation weakens the skin barrier, allowing more stimulus to penetrate and trigger PPIs that result in immune reactions capable of eradicating the stimulus and also lead to inflammation (figure 1*a*). Furthermore, our model allows us to identify particular terms and parameters that disrupt these balances at the cellular and tissue levels, which naturally correspond to well-known AD risk factors.

Our model reproduces several key clinical features of AD. In particular, it clarifies the relationship between risk factors and specific dynamical responses to external stimuli. The model also predicts, in a quantitative manner, the increase in the susceptibility of the epidermis to developing atopic conditions given multiple risk factors. These results contribute to the identification of the key underlying regulatory interplay between skin barrier homeostasis and inflammation in AD, and may lead to a more accurate characterization of the disease symptoms required for personalized treatment. Moreover, our modelling approach, based on the time-scale separation and the view that the disease occurs as a result of loss of balance between dual (positive and negative) feedbacks, provides a theoretical framework for the study of multi-scale regulatory interactions in many other physiological systems. We discuss the generality of our modelling approach for studying the regulatory interactions between the cellular-level inflammatory response and tissue-level regulation of epithelial homeostasis.

## 2. Multi-scale model for atopic dermatitis

Our multi-scale model of epidermal homeostasis builds upon a recent cellular-level model for regulation of KLK activities [22], which we couple to a model of tissue-level regulation. The input and output of the cellular-level model are the stimulus and the resultant inflammation, respectively, which, in turn, become the output and input of the tissue-level model (figure 1*c*). The biological processes considered in the multi-scale model are explained below, while a complete model description, together with the nominal parameters used in the simulation, is found in appendix A. The corresponding processes in the model (figure 1*a*) are indicated by the same labels (a)–(f) in figure 1*c*.

### 2.1. Modelling the tissue-level processes

Healthy skin with high barrier integrity prevents exacerbated penetration of environmental stimuli into the inner epidermal layers (figure 1*a*(g)) [23]. Our model considers the skin barrier integrity determined by a combination of the amount of corneocytes [24], their cohesion [25] and the lipid content [1] in the skin barrier. Accordingly, high barrier integrity is preserved by an appropriate balance between the production and desquamation rates of corneocytes and by maintenance of high lipid content. Skin barrier integrity is weakened as a result of cellular-level responses. Excessive activation of KLKs leads to increased skin barrier desquamation by degrading the inter-cellular junctions (figure 1*a*(a)) [24], while activation of protease-activated receptor 2 (PAR2) by active KLKs (figure 1*a*(c)) leads to enhanced inhibition of the lamellar body lipid release into the skin barrier (figure 1*a*(b)) [5]. Upon disruption, the skin barrier triggers self-restoring mechanisms, in the forms of gene expression, lipid release and transition of terminally differentiated keratinocytes to corneocytes [1,5,6].

A defective skin barrier with low barrier integrity allows more exogenous stimuli to invade the inner epidermal layers (figure 1*a*(d)), forming a positive feedback loop from active KLK and PAR2 (denoted by 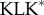 and 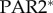 hereafter) to the stimulus concentration. For the inflammatory states, a large amount of 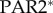 is induced and internalized, which then transduces stronger canonical signalling cascades and increases the expression of pro-inflammatory genes (figure 1*a*(e)) [26]. The inflammatory level in our model is accordingly represented by the level of 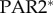. These signalling cascades also trigger 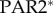-mediated immune reactions which persist even after the inactivation of 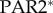 [27]. 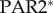-induced immune reactions eradicate the accumulated stimulus in the inner epidermal layers (figure 1*a*(f)) by mediating the release of antimicrobial peptides or the induction of keratinocyte phagocytosis [1,28], forming a negative feedback loop from 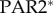 to the stimulus concentration. The concentration of stimulus that penetrates the inner epidermal layers is thus determined by the balance between the positive and negative feedback regulations, whose strengths, respectively, depend on the skin permeability and the capacity of stimulus eradication (figure 1*d*).

The activities of the KLKs are regulated by the cellular-level PPI network (figure 1*b*), which is induced by the internalized stimulus, as modelled by Tanaka *et al.* [22]. Our model considers infiltrated stimuli, such as *Staphylococcus aureus*, which promote the production of KLK via the activation of pattern-recognition receptors [29,30]. Stimulus-triggered release of KLK and its inhibitor LEKTI into the extracellular space (figure 1*b*(a)) is followed by auto-activation of KLKs (figure 1*b*(b)), inhibition of 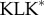 by LEKTI (figure 1*b*(c)), and activation of PAR2 through 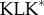 by proteolysis (figure 1*b*(d)). All of these protein interactions occur in a pH-dependent manner. The model also includes positive feedback from 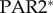 to the expression of KLKs and LEKTI (figure 1*b*(e)). The activities of the KLKs are thus determined by the balance between their activation and inhibition rates (figure 1*d*).

We represent this multi-scale system of regulatory interactions as a system of integral–differential equations with the time-scale separation by assuming that the steady states of the cellular-level enzymatic reactions (attained in seconds to minutes [31]) affect the tissue-level changes in the skin barrier (in hours after perturbation by the external stimulus [5]).

### 2.2. Modelling the effects of risk factors for atopic dermatitis

We consider four risk factors that break the balanced regulation at each level, that is, the balance between the activation and inhibition of 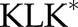 at the cellular level and the balance between the positive and negative feedback controls at the tissue level. They correspond to the following four main genetic and environmental elements known to predispose persons to AD.

*High pH*. Increased pH in the epidermis (pH around 6.5 in AD patients compared with 4.5 in healthy skin) leads to increased catalytic activity of KLKs [8,24].
*Low LEKTI*. A decrease in the basal expression rate of LEKTI, a KLK inhibitor, leads to compromised inhibition of KLK activity [12].
*High permeability*. Increased skin barrier permeability, for example by a genetically determined decrease in the expression of filaggrin (an essential protein for maintaining strong cohesion between epithelial cells) [25], allows increased penetration of stimulus, leading to strong positive feedback from 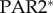 and 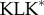 to the stimulus concentration. It is modelled with increased nominal skin permeability, *P̃*, which corresponds to the permeability of the unaffected skin.
*Weak stimulus eradication*. Decreased capacity of the immune reactions to eliminate the infiltrating stimuli [32,33] leads to compromised negative feedback from 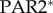 to the stimulus concentration.

We investigate the effects of the risk factors (in isolation and combination) on the development of AD by altering the corresponding parameters in the model. The parameters corresponding to the cellular-level risk factors, ‘high pH’ and ‘low LEKTI’, are taken from Tanaka *et al.* [22]. New parameters corresponding to the tissue-level risk factors, ‘high permeability’ and ‘weak stimulus eradication’, are varied to see the effects of the balance between the positive and negative feedbacks on the development of AD.

## 3. Bistable switch with hysteresis at the cellular level

We are interested in the steady states of 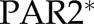 and 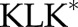 (denoted hereafter as 
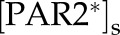
 and 
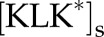
, with the subscript s indicating steady states), since we assume that 
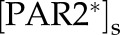
 and 
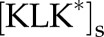
 are reached quickly compared with the overall tissue dynamics which they affect.

**Figure 2.**
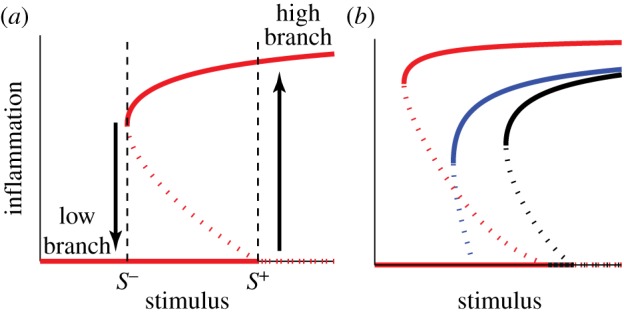
Cellular-level switch-like behaviour. Adapted from Tanaka *et al.* [22]. (*a*) Dose–response behaviour of PAR2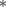 (inflammation) represented by bistability in the bifurcation diagram with varying concentrations of stimulus. The inflammation is on the low branch (no inflammation) until the stimulus increases to the inflammatory threshold *S*^+^, at which point the inflammation switches to the high branch and persists until the stimulus falls below the recovery threshold *S*^−^. (*b*) Cellular-level risk factors increase the severity of inflammation compared with the healthy condition (black). ‘Low LEKTI’ (blue) decreases *S*^+^, increasing the sensitivity to the stimulus. ‘High pH’ (red) decreases *S*^−^, resulting in more sustained inflammation.

Figure 2*a* shows the bifurcation diagram for 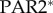 (inflammation) described by Tanaka *et al.* [22] with the inter-epidermal stimulus concentration as a bifurcation parameter. This dose–response curve exhibits bistability, with *low* and *high branches* corresponding to the non-inflamed and inflamed states, respectively. Therefore, the state switches to the high branch when the stimulus increases above the *inflammation threshold* *S*^+^ and remains there until the stimulus decreases below the *recovery threshold* 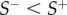, at which point it goes back to the low branch and the inflammation stops. This classic hysteretic behaviour induced by the bistability of the model captures the characteristic features of AD, namely the outbreak and persistence of inflammation [22]. We note that similar bistability and hysteresis is observed for 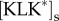 with the same *S*^+^ and *S*^−^ values as for 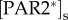.

The effects of two risk factors, low LEKTI and high pH, on the development of AD have been investigated by Tanaka *et al.* [22], and they are in good agreement with clinical data from AD patients. Both risk factors increase the severity of the inflammation (figure 2*b*) but in different ways: a low LEKTI condition (blue) results in a lower *S*^+^, corresponding to more susceptibility to the stimulus, as it requires less stimuli for inflammation to occur. A high pH condition (red) leads to a more persistent inflammation with lower *S*^−^, as it requires a further decrease in the stimuli for the inflammation to stop.

## 4. Dynamical behaviours in the multi-scale model

This section presents a numerical characterization of the slow, tissue-level dynamical behaviour of the full system in response to external stimuli, especially the dynamical behaviour of the skin barrier integrity, *B*, and the inter-epidermal stimulus concentration, *S*, which was a bifurcation parameter in the previous section. They both depend on 
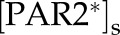
 and 
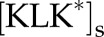
 (figure 1*c*, equation (A 1)). The skin barrier integrity is a clinically relevant variable as an indicator of epidermis health and can be measured by non-invasive methods. We can also follow the dynamic behaviour of 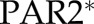-mediated inflammation, which is induced when the barrier is damaged and ceases when the barrier starts to recover in the absence of 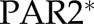.

All the simulations presented below correspond to the response of the system to an external stimulus in sufficiently large concentration such that *S* rises above the inflammation threshold *S*^+^, moving the system onto the high branch in the bifurcation diagram for both cellular-level risk factors with a nominal barrier permeability (figure 2*b*).

### 4.1. Classification of dynamical behaviours in the slow time

**Figure 3.**
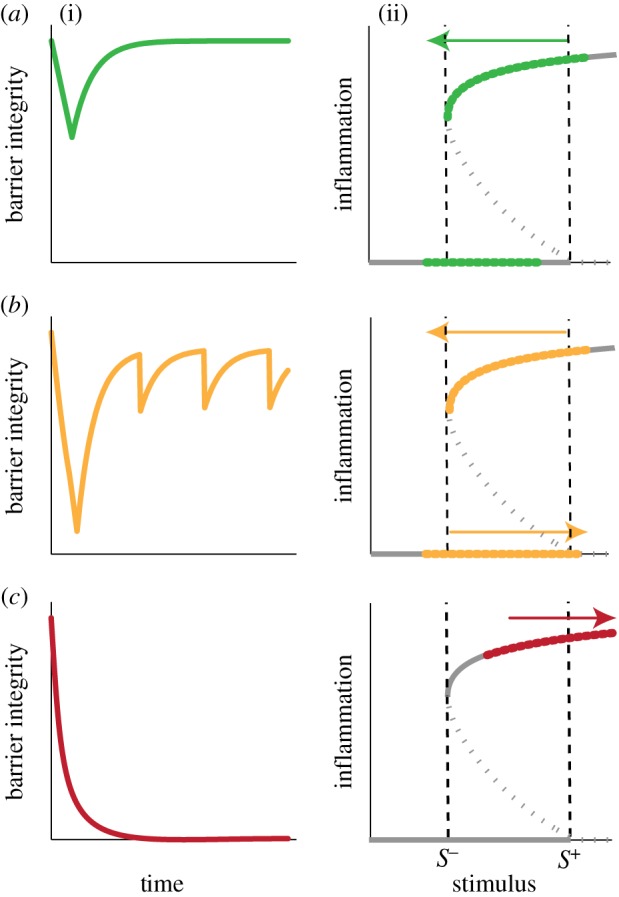
Three qualitative dynamic behaviours of skin barrier integrity after environmental challenges. (*a*) Homeostasis: observed when the stimulus is decreased to *S*^−^ and the inflammation stops. (*b*) Oscillation: observed when the stimulus is repeatedly decreased to *S*^−^ (inflammation stops) and then re-increased to *S*^+^ (inflammation recurs). (*c*) Persistent damage: observed when the stimulus fails to decrease to *S*^−^.

For different sets of parameters, our model exhibits three qualitatively different dynamic behaviours for the skin barrier integrity when responding to environmental challenges of the stimulus (figure 3), in order of increasing severity of the AD manifestation:

*Homeostasis*. Complete recovery to the homeostatic level, typical for a healthy epidermis,
*Oscillation*. Periodic loss of homeostasis, often found in moderate AD skin, and
*Persistent damage*. Incapability of recovery to the homeostatic level, often observed in severe AD skin.

These three behaviours can be understood in terms of the interplay between the slow and fast dynamics leading to a quasi-static sweep of the bifurcation curve of the fast system.

In the case of homeostasis, the stimulus *S* is eradicated via immune reactions and falls below the recovery threshold *S*^−^, at which point the inflammation stops as it jumps to the low branch (figure 3*a*(ii)). The barrier integrity then recovers to its homeostatic value with no inflammation (figure 3*a*(i)). This behaviour is observed when the stimulus eradication is strong enough to decrease *S* below *S*^−^ through negative feedback, although the skin barrier suffers from transient damage while the inflammation is on the high branch, leading to the increase in *S* through positive feedback.

In some cases, however, *S* can increase again to *S*^+^ after it transiently falls below *S*^−^, thus re-triggering inflammation that again decreases *S* below *S*^−^ through the same mechanism. This cycle leads to an oscillation of the skin barrier integrity (figure 3*b*). Whether *S* re-increases to *S*^+^ or not is determined by its steady-state concentration, *S*_s_, when the inflammation is on the low branch (no inflammation). If *S*_s_ is higher than *S*^+^, then *S* eventually exceeds *S*^+^. A high *S*_s_ reflects a weak barrier which cannot keep *S* low enough without the inflammation causing negative feedback on *S*.

Under other conditions, *S* cannot be decreased below *S*^−^; for example, when the stimulus eradication is not strong enough (weak negative feedback) or when the barrier permeability is too high (strong positive feedback; figure 3*c*). Such conditions lead to persistent damage of the skin barrier integrity with the complete loss of barrier integrity, as the inflammation cannot be resolved.

In addition to the qualitative nature of the dynamical behaviour, the severity of the transient barrier damage can be quantitatively evaluated in terms of two clinically relevant measures: the recovery time required for the barrier integrity to recover to its homeostatic level and the maximum amplitude of the damage to the skin barrier. These two measures are usually not independent: the maximum amplitude of the barrier damage depends on the recovery time, since the longer the recovery takes, the heavier the damage is to the barrier.

### 4.2. Risk factor dependence of dynamical behaviours in the slow time

**Figure 4.**
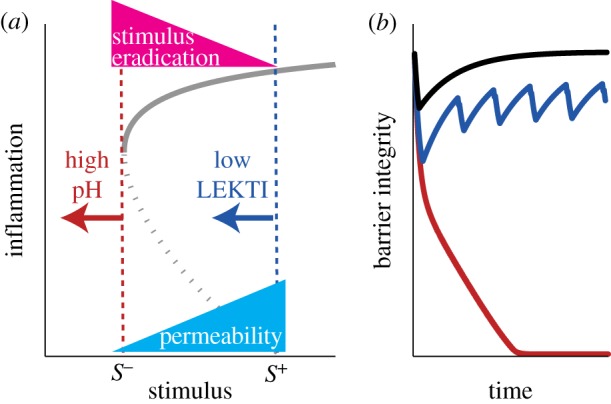
Effects of risk factors. (*a*) Effects of single risk factors on the cellular-level bifurcation diagram. ‘High pH’ decreases *S*^−^ and ‘weak stimulus eradication’ prevents the decrease of *S*, resulting in difficulty in lowering *S* below *S*^−^. ‘Low LEKTI’ decreases *S*^+^ and ‘high permeability’ increases *S*, facilitating *S* to go above *S*^+^. An increase of *S* by ‘high permeability’ makes it more difficult to reach *S*^−^. (*b*) Risk factor-dependent behaviour of barrier integrity. While healthy (black) shows homeostasis, ‘high pH’ (red) leads to persistent damage, whereas ‘low LEKTI’ (blue) results in oscillation. The effects of the tissue-level risk factors are shown in figure 5.

Our model predicts the risk factor dependence of the observed behaviour for the skin barrier integrity classified above, based on the different effects of each risk factor on the tissue-level dynamics of *S*, the value of *S*_s_, which is a solution of the multi-scale model, and the threshold values *S*^+^ and *S*^−^ determined by the cellular-level PPI (figure 4*a*).

Since the low LEKTI condition leads to a decrease in the inflammatory threshold *S*^+^ (figure 2*b*, blue), *S* is more likely to increase to *S*^+^ after it transiently decreases below *S*^−^, thereby leading to an oscillation of the epidermal integrity (figure 4*b*, blue). The high pH condition with a lower recovery threshold *S*^−^ (figure 2*b*, red) leads to persistent damage (figure 4*b*, red), because it is more difficult to decrease *S* below *S*^−^.

**Figure 5.**
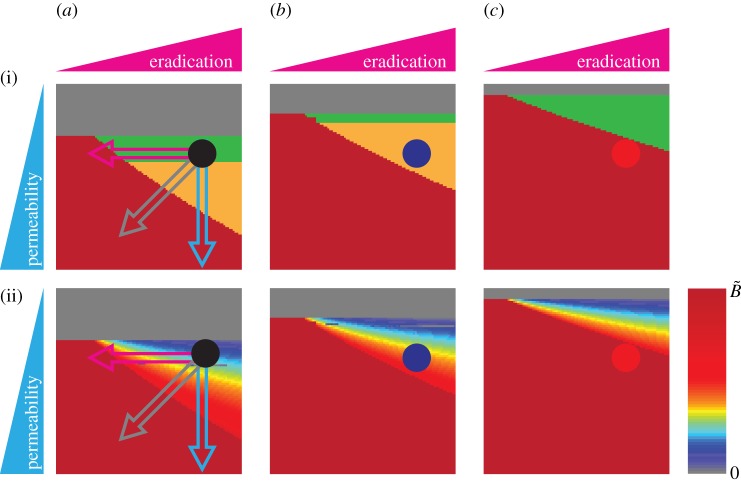
Increase in skin vulnerability caused by the presence of multiple risk factors. The figure presents a summary of the bifurcations and dynamical behaviours observed for different combinations of tissue-level risk factors (‘high permeability’ and ‘weak stimulation eradication’) for (*a*) healthy (high LEKTI and low pH), (*b*) low LEKTI and (*c*) high pH conditions. The black, blue and red circles in (*a*), (*b*) and (*c*), respectively, correspond to the nominal values used for the simulation in figure 4*b*. Increase in AD severity is represented (i) by transition of the dynamical behaviours of the epidermal integrity from homeostasis (green) to oscillations (yellow) and further to persistent damage (red) accompanied (ii) by increases in the maximum amplitude of the skin damage. The grey area indicates ‘non-responsive’ owing to the strong skin barrier integrity. (*a*) Increasing permeability (blue arrow) leads to a qualitative transition from homeostasis to oscillation, and further to persistent damage, accompanied by a continuous increase in the maximum amplitude. Decreasing the capacity of stimulus eradication (pink arrow) results in a direct transition from homeostasis to persistent damage. Concurrence of the two tissue-level risk factors accelerates this transition (grey arrow). Additional presence of cellular-level risk factors (‘low LEKTI’ (*b*) or ‘high pH’ (*c*)) increases the skin vulnerability with a smaller region of ‘non-responsive’ and ‘homeostasis’ but with a larger region of ‘oscillation’ and ‘persistent damage’, compared with healthy (*a*).

The high permeability increases *S* by allowing more of the external stimulus to penetrate (figure 4*a*). The larger *S* is, the more easily *S*^+^ is reached, leading to the oscillation, and the more difficult *S* is decreased to *S*^−^, leading to the persistent damage. Increasing the degree of the high permeability risk factor (figure 5*a*, blue arrow) leads to a qualitative transition from homeostasis (green) to oscillation (yellow), and further to persistent damage (red). This transition is accompanied by a continuous increase in the maximum amplitude of the barrier damage. Note that the external stimulus does not penetrate enough to induce the inflammatory response (‘non-responsive’) if the permeability is very low (figure 5*a*, grey).

When the capacity of stimulus eradication is weak, *S* cannot be decreased even with high inflammation (figure 4*a*). Decreasing the capacity of stimulus eradication (figure 5*a*, pink arrow) results in a qualitative transition from homeostasis (green) to persistent damage (red) directly, with a continuous increase in the maximum amplitude of barrier damage. Weakening the stimulus eradication capacity by itself does not lead to the onset of oscillation, since it does not affect either of the two oscillation-determining factors, i.e. *S*_s_ (determined by the non-inflamed state) or *S*^+^ (determined by the cellular-level PPI).

Our quantitative analysis of the proposed model suggests that the severity of the (transient) damage to the skin barrier is significantly increased by the concurrence of multiple risk factors, as is often the case for AD [11,25,34]. Concurrence of the two tissue-level risk factors (‘high permeability’ and ‘weak eradication’) makes the transition from homeostasis to oscillation and persistent damage occur by a smaller increase in the degree of each risk factor, resulting in more frequent occurrences of oscillation and persistent damage with a larger maximum amplitude of barrier damage (figure 5*a*, grey arrow). Additional presence of cellular-level risk factors (‘low LEKTI’, figure 5*b*, or ‘high pH’, figure 5*c*) further increases the skin vulnerability, with a lower occurrence of ‘non-responsive’ (grey) and ‘homeostasis’ (green) and a higher proclivity towards ‘oscillation’ (yellow) and ‘persistent damage’ (red) with a larger maximum amplitude of barrier damage.

## 5. Discussion

### 5.1. The regulatory structure of the model leads to distinct dynamic signatures for different risk factors

We have proposed a multi-scale model of the epidermis as a means to investigate the regulatory interplay between the tissue-level skin barrier integrity and cellular-level inflammation with an intrinsic separation of time scales. As a result of a combination of the cellular-level switching and the tissue-level feedback control (figure 1*d*), our model displays three qualitatively different dynamic responses for skin barrier integrity: homeostasis, oscillations and persistent damage (figure 3). These behaviours emerge from a combination of balanced feedback regulation at the cellular and tissue levels.

Our multi-scale model allowed us to assess the impact of several risk factors on the epidermal function and suggests that each risk factor has its own specific dynamic signature (figure 5). These features could be used to characterize patient-specific causes of AD from the observed dynamic behaviours of the epidermis and to improve the personalized treatment of AD. Importantly, the risk factor-dependent qualitative behaviours do not depend on the particular choice of system parameters, but rather on the general structure of the multi-scale regulatory network model and how the different terms are affected by the risk factors. The 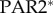-induced inflammation is regulated by a dual (positive and negative) control at the cellular level: negative control with inhibition of 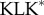 by LEKTI and positive control with pH-dependent activation of 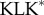 and 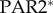. The concentration of stimulus at the tissue level is also controlled in a dual way: negative control to decrease it through its eradication and positive control to increase it by degrading the skin barrier. A balance between the two dual controls acting at the cellular and tissue levels is essential to maintaining the healthy homeostasis. The risk factors in this paper correspond to disturbed feedback strengths that cause an imbalance of the dual control (figure 1*d*), leading to either oscillatory or persistent loss of homeostasis. The concurrence of multiple risk factors, each of which affects the dual control, dramatically increases the fragility of the regulatory system of epithelial function, even if the system is robust enough to buffer out disturbances caused by single risk factors.

### 5.2. Applicability to other inflammatory diseases

One of the main features of our model is the presence of a switch at the cellular level realized by the bistability in the bifurcation diagram of the inflammation (figure 2*a*), coupled with a slower regulatory system. This structure makes our model resemble an ‘on–off’ hybrid system [35], in which switching between two dynamic control regimes (depending on whether the inflammation is ‘on’ or ‘off’) determines the global behaviour of the system.

Pathogen-induced signalling cascades play a key role in protecting the body from infection by triggering immune reactions that contribute to the eradication of the stimulus. However, these signalling cascades also trigger tissue-damaging inflammation, since the induction of stimulus-eradicating mechanisms often concurs with the release of pro-inflammatory mediators [36–38]. The switch mechanism prevents unnecessary and inefficient immune reactions to be triggered, since the activation of the signalling cascades occurs only when the stimulus concentration is high enough (above the inflammation threshold) to endanger the tissue, and the immune reactions persist until the stimulus falls significantly below the inflammation threshold. This ensures that inflammation does not recur through random fluctuations of the stimulus concentration.

Unnecessary and ineffective immune reactions are accompanied by inflammation that can cause the destruction of tissues and has a significant impact on the epithelial function by affecting the morphology [4,28], composition [39], abundance [40] and micro-environment [1] of epithelial cells, without contributing to eradicating pathogens [41,42]. Indeed, this constitutes a major part of pathology in many inflammatory and infectious conditions, including AD [41–43]. Therefore, fast resolution of the inflammation is essential for the restoration of homeostasis, and relies on a balance between negative (via stimulus eradication) and positive (via tissue damage) feedback from the inflammation to the stimulus concentration.

The bistability of the cellular-level inflammatory responses together with the dual feedback on a longer time scale constitute a biologically reasonable architecture in terms of efficiency and robustness, and it has been found in many inflammation-inducing PPI networks [44,45]. For example, bistability has been found in the multi-scale regulatory networks of the inflammatory response induced by cytokine interleukin 1 (IL1), another system of interest as regards AD [13,46]. This system is similar in structure to the KLK system discussed in this paper, since PPI leading to IL1 activation is also triggered by stimuli that infiltrate through the skin barrier [47], including several biotic and abiotic allergens [38,48], and active IL1 affects the stimulus concentration by initiating immune reactions that eliminate the stimulus [38] and by altering skin barrier formation [49]. Our model can also be applied to the other two atopic diseases, asthma and hay fever, since the regulation of bronchial and nasal epithelium function occurs though a similar interplay between the tissue-level barrier and cellular-level inflammatory response in the epidermis, bronchial epithelium and nasal epithelium [9,14].

### 5.3. Clinical significance

Despite its clinical importance, the epidermal function has seldom been studied from a systems-level perspective. Conventional experimental approaches have not fully revealed the relationships between the barrier dysfunction and inflammatory response in the development of AD [33,50]. Our model provides a platform to analyse the bi-directional interplay between skin barrier dysfunction and aberrant inflammatory response (figure 1*d*).

Our model suggests that the measurement of the barrier integrity over time can predict the type of clinical course a particular patient is expected to follow (figure 3). This is also compatible with clinical findings: the long-term prognosis of atopic patients may be related to the type of behaviour observed; whereas persistent inflammation is associated with a poor prognosis, patients with periodic inflammation have a better chance for resolution of the disease, indicating that there may be a correspondence between these behaviours and increasing severities of atopic diseases [51].

An empirical validation of our model predictions, particularly the association between the four risk factors and dynamic model behaviours (figure 5), and implementing the prediction in dermatological practice will require an empirical system-level investigation of the epithelium to measure the barrier integrity and inflammation over time for different phenotypes and environmental conditions. For this purpose, it is essential to develop a new way to continuously measure the barrier integrity over several hours to a day; for example, by non-invasive measurement of transepidermal water loss and skin hydration measurements in AD patients [52] or by use of organotypic cultures, which allow temporal and spatial quantitative characterization of the skin [53]. Validation of our results through such studies will contribute to a more efficient, patient-specific treatment of AD and other atopic diseases, since the risk factor triggering the disease condition in a particular patient can be deduced from the time course of the barrier integrity.

### 5.4. Possible extensions of the model and future work

Our model considers the essential processes and reactions that contribute to the regulatory interplay between tissue- and cellular-level dynamics at different time scales from the viewpoint of dual controls. This framework could be extended to include another level with an even slower time scale corresponding to the proliferation and differentiation of epithelial cells. The regeneration of the skin barrier requires skin barrier precursors, i.e. correctly differentiated cells at the lower layers of the epithelium [54]. Our model assumes a constant concentration *b*_pre_ of skin barrier precursors. However, formation of these barrier precursors is in fact a dynamic process that is orchestrated among differentiation, proliferation and apoptosis of epithelial cells, and is further affected by inflammatory signals [49,55]. Describing such dynamics of the epithelial cells at an even slower time scale and coupling it to the multi-scale model proposed here would allow us to explore the interplay between inflammation and epithelial tissue renewal, and to address interesting, open questions regarding deregulation of epithelium renewal in the context of inflammation, which plays an important role not only for AD [54] but also for the development of tumours [56].

The skin immune system can interact with the systemic immune system in AD, which may be incorporated into the model in the future. It has been widely observed that AD patients are more prone to develop asthma and hay fever, leading to the so-called atopic march [14,57]. It is thought that allergic reactions occur in different organs owing to *allergic sensitization*, which is the induction of allergic reaction and the establishment of immunological memory by repeated exposure of inter-epithelial cells to antigen [14]. This sensitization usually occurs at the systemic level, and the exposure to the allergen in other organs, after the establishment of sensitization in the skin, can initiate allergic reactions and subsequent immunopathology there, although local factors such as the integrity of epithelium may also be important for the development of allergic diseases [58]. Incorporation of the allergic reaction in the model would allow us to explore not only the mechanisms of the atopic march but also the allergic diseases of strong clinical relevance [59,60], while our model considers early events in the pathogenesis of AD that occur before adaptive immune responses to the stimuli, such as histamine release by primed mast cells, are initiated.

### 5.5. Concluding remarks

We proposed a multi-scale model for epithelial function which predicts that the underlying risk factors for AD can be inferred from the clinically observable dynamic response of the skin barrier to environmental challenges. Our model analysis using time-scale separation reveals the effects of AD risk factors on the epidermal function regulated by the dynamic interplay between inflammation and skin barrier permeability. Our results may lead to a better understanding of the design principles of the regulatory systems for epithelial homeostasis and inflammation and may be able to be extrapolated to other atopic diseases. Our modelling approach provides a theoretical framework for studying multi-scale regulatory interactions, characteristic of nearly all physiological systems.

Atopic diseases represent an important and unresolved health problem, mainly because their causes have not yet been elucidated owing to the complex nature of the underlying regulatory networks across different scales. Clinical research will benefit from our systems-level mathematical framework for studying epithelium function, as it will allow systematic evaluations of the effects of risk factors and different treatments, such as corticosteroids or emollients, on clinically observable epithelium markers such as the barrier function. We expect that our research will contribute to a deeper understanding, more accurate description and patient-specific treatment of atopic diseases.

## Appendix A

### A.1. Multi-scale kallikrein model description

Our multi-scale model is a combination of the cellular-level model proposed by Tanaka *et al.* [22] (model 1 with positive feedback from  to LEKTI production) and a tissue-level model described as

A1a

and

A2b

where *τ* corresponds to the slow time scale, *S* is the concentration of the inter-epidermal stimulus and *B* is the barrier integrity.

The inter-epidermal stimulus, *S*, increases by the penetration of external stimulus, *S*_out_, through the barrier, hence its rate is proportional to *S*_out_ and inversely proportional to the skin barrier integrity, *B* (figure 1*c*(d)). The minimum barrier integrity, , reflects the fact that even very severe cases of AD [61] do not lead to a complete destruction of the skin barrier and ensures that the model is always well defined. Eradication of *S* occurs by the combination of -independent (*I*_0_) and -dependent (*I*_P_) immune reactions (figure 1*c*(f)) represented by

A3

where the convolution with a decaying exponential captures the persistence of the immune reactions even after inactivation of  [27].

The barrier integrity, *B*, is determined by the balance between the production and degradation of the barrier. The barrier is produced from precursors, which are assumed to be constantly available with a fixed concentration, *b*_pre_, and lipids, whose release is inhibited by  (figure 1*c*(b)). The capacity of the barrier to self-restore the nominal barrier integrity, *B̃*, following its disruption [1,6] is represented by a logistic term. The degradation of the barrier occurs as a result of desquamation mediated by  (figure 1*c*(a)).

Note that  and  are the stable steady-state concentrations of  and  obtained from the ODE model [22] for the cellular-level PPI reaction network which describes the dynamic behaviour of the concentration of six species, including  and , as a six-dimensional system of ODEs with 21 parameters.

### A.2. Parameter values and robustness of the model outcomes

The nominal values of the parameters for the cellular-level model are taken from Tanaka *et al.* [22]. For the tissue-level model, we chose nominal values (table 1) that are consistent with available experimental data. Since the experimental data to date are qualitative rather than quantitative, our model predictions are concerned with the qualitative effects of the risk factors, in terms of a loss of balance of the dual control, on the system's behaviour.

**Table 1.** Definitions of systems parameters and their nominal values used for the simulations.

parameter	description	nominal value
*S*_out_	concentration of external stimulus	95
*P̃*	nominal skin permeability	0.4
*f*_IP_	strength of stimulus eradication by PAR2*-mediated immune reactions	0.8
*I*_0_	PAR2*-independent immune reactions	1
*b*_pre_	concentration of skin barrier precursors	0.5
*k*_L_	strength of lipid release inhibition by PAR2*	10
*B̃*	nominal skin barrier integrity	1
*d*_K_	rate of desquamation by KLK*	0.1
	minimum barrier integrity	0.01

Such results are largely robust to the parameter choices, as confirmed by a detailed analysis of the sensitivity of the parameters on the model behaviours. Our numerics show that the qualitative behaviour is preserved when the parameters are changed 10–100-fold. More specifically, the -independent immune reactions (*I*_0_) and the nominal skin barrier integrity (*B̃*) are scaling factors for *S*_s_. The concentration of skin barrier precursors (*b*_pre_) affects the skin barrier recovery time, but does not alter the qualitative behaviours. The strength of lipid release inhibition by  () and the rate of desquamation by  () scale the concentrations of  and , thus affecting the propensity for persistent loss of homeostasis but not the qualitative behaviours.

The other two parameters of the model encapsulate the effects of particular risk factors. The skin permeability (*P̃*) was varied from  (no influx of the stimulus) to *P̃* = 1 (100% permeable barrier) to assess the effects of the ‘high permeability’ risk factor on the model behaviours (figure 5). The effects of the ‘weak stimulus eradication’ risk factor were assessed through varying the corresponding parameter (*f*_IP_) from 0.01 (weak stimulus eradication) to 1 (healthy stimulus eradication; figure 5). Varying *f*_IP_ for orders of magnitude higher and lower did not show qualitatively different results.

### A.3. Model analysis

and  were calculated by solving the fixed point for algebraic equations of the cellular-level model [22] using Maple v. 13 (Maplesoft, Waterloo, Ontario, Canada). These were used for the dynamic simulations of the multi-scale model using the ODE solver *ode23t* in Matlab v. R2010a (The MathWorks, Inc., Natick, MA, USA). At each iteration of the solver, the values of  and  were updated according to the history of *S*, by using the *odeset* command in the function declaration.
